# Assessing the Efficacy of Albendazole against *Fasciola hepatica* in Naturally Infected Cattle by In Vivo and In Vitro Methods

**DOI:** 10.3390/vetsci8110249

**Published:** 2021-10-25

**Authors:** Michal Babják, Alžbeta Königová, Ľudmila Burcáková, Michaela Komáromyová, Michaela Urda Dolinská, Marián Várady

**Affiliations:** Institute of Parasitology, Slovak Academy of Sciences, Hlinkova 3, 040 01 Košice, Slovakia; babjak@saske.sk (M.B.); burcakova@saske.sk (Ľ.B.); komaromyova@saske.sk (M.K.); dolinska@saske.sk (M.U.D.)

**Keywords:** *Fasciola hepatica*, albendazole, anthelmintic resistance, cattle

## Abstract

This study was performed on a cattle farm with a long-term use of albendazole (ABZ) and a permanent history of fasciolosis for comparing in vivo and in vitro methods for the detection of anthelmintic resistance and drug efficacy. A selected group of 10 Charolais cows was treated in autumn 2020 with ABZ at a dose of 7.5 mg/kg body weight. Another group of 10 cows remained untreated as a control. The faecal egg count reduction test was used to determine in vivo efficacy. The percentage reduction of eggs on day 14 after treatment ranged from 77 to 81.8%, depending on the formula used for calculation. The in vitro egg hatch test (EHT) was used as a second diagnostic method. *F. hepatica* eggs for the EHT were isolated from faecal samples. The test was performed in two versions differing in the length of incubation with ABZ (12 h and 15 d). The percentage of eggs with inhibited development at a concentration of 0.5 μM in both versions of the EHT agreed with the in vivo results. Ovicidal activity at a concentration of 0.5 μM in the 12-h version suggested a reduced efficacy of ABZ (65.40%). An EHT prepared using pooled faecal samples was a prospective method for the detection of efficacy and ABZ resistance in *F. hepatica*.

## 1. Introduction

*Fasciola hepatica* is the most globally widespread helminth parasite [1]. This common liver fluke can infect a wide range of hosts and is responsible for substantial losses, mainly in the production of grazing ruminants [2]. The emergence of anthelmintic resistance (AR) is well known mainly in gastrointestinal parasites on small-ruminant farms. Similar economic and health risks can pose development of AR in *Fasciola hepatica* even more that represents a threat as zoonosis [3,4], and a case of triclabendazole (TCBZ) resistance has already been confirmed in human infection [5]. Triclabendazole is still the first choice for the treatment of fasciolosis due to its efficacy against mature and immature flukes [6]. The first case of AR in liver flukes against TCBZ was described in sheep by Overend and Bowen [7] in Australia. Numerous cases of TCBZ resistance have since been reported worldwide on small-ruminant and cattle farms, which have been summarised by Kelley et al. [8] and McMahon et al. [9].

Albendazole (ABZ) presents another option for the treatment of fasciolosis but only has limited anthelmintic activity against adult flukes older than 12 weeks [10,11]. Coles and Stafford [12] reported that ABZ reduced *F. hepatica* adults in a TCBZ-resistant isolate by 95%. The trend in the incidence of AR in *F. hepatica*, due to the frequent use of ABZ on ruminant farms, however, can be assumed to be increasing. Cases of ABZ resistance on sheep farms have been commonly reported from South America [13,14,15] and Europe [16,17,18]. ABZ resistance in *F. hepatica* in cattle has been described in Turkey [19], Peru [20], Egypt [21] and Tanzania [22]. More than 20 years have passed since the first reported case of AR in *F. hepatica*, but standardised protocols for identifying the efficacy of new drugs and for detecting AR are still not available [1]. A controlled efficacy test is the most accurate method, based on post-mortem counts of flukes after therapy in treated groups compared to control groups [12,23]. This method, however, is used only rarely due to economic and time-consuming reasons.

Diagnosis using an in vivo faecal egg count reduction test (FECRT) is more limited than in gastrointestinal parasites due to possible false positive results when eggs are stored in the gallbladder, even when adults are removed after effective treatment [24,25]. False negative results can also be due to intermittent egg output, which begins up to several months after infection [26]. Verifying and comparing the results of in vivo efficacy with in vitro or molecular methods would therefore be appropriate. A coproantigen reduction test (CRT), which has been successfully used in several studies in naturally infected sheep and cattle [27,28,29], may be an applicable method. The use of several complementary methods for the detection of AR was suggested by Hanna et al. [30], where diagnosis on sheep farms using an FECRT was supported by CRT and fluke histology.

The in vitro egg hatch test (EHT) based on the activity of some benzimidazole (BZ) compounds against *F. hepatica* eggs is another possibility for diagnosis [31]. Canevari et al. [32] characterised the EHT as a suitable method for detecting resistance in *F. hepatica* after the test reliably distinguished between susceptible and resistant isolates. The EHT was successfully used as a complementary method in Sweden for the detection of ABZ resistance on sheep farms [18]. Alvarez et al. [33] described two versions of the test that differed in the length of time fluke eggs were exposed to an anthelmintic.

Field studies of naturally infected animals should be an integral part of the standardisation of tests for the detection of resistance in flukes. The main goals of this study were to: (i) determine the efficacy of ABZ against *F. hepatica* in farmed cows with long-term use of ABZ, (ii) compare the results of in vivo and in vitro methods and (iii) compare the results between two versions of the EHT.

## 2. Materials and Methods

### 2.1. Animals, Farm and Study Design

The study was carried out on a farm in northeastern Slovakia with a long-term use of ABZ and a permanent history of fasciolosis. The herd consisted of 300 Charolais cows reared for meat production. The cattle spend most of the year on pastures, from early spring to late autumn. The pastures were near a water source, and wetland often formed after heavy rains in summer.

All animals were housed in autumn 2020, and a selected group of 10 cows was treated with a dose of ABZ (7.5 mg/kg body weight (bw), Albendavet 10%^®^, DIVASA-FARMAVIC S.A., Barcelona, Spain). Another group of 10 cows remained untreated as a control. These animals had not been treated with any anthelmintic for one year prior to the experiment. Faecal samples were individually collected on day 0 (D0) and D14 after treatment. Part of the samples collected on D0 was used as a pooled sample in the EHT.

### 2.2. Fluke Egg Detection

Fluke eggs were collected using the method described by Graham-Brown et al. [34]. The composite sample consisted of 10 individual 10-g samples (100 g). A 10-g subsample was subsequently used for sedimentation. The subsample was homogenised in tap water and filtered through a set of three sieves of different mesh sizes (250, 100 and 50 μm). The part of the sample that remained on the last sieve was transferred to a 200-mL glass beaker, diluted to a volume of 100-mL and let stand for 5 min to allow the sedimentation of *F. hepatica* eggs. After 5 min, the supernatant was discarded, and the sediment was again diluted to a volume of 100 mL. This process was repeated until the suspension was completely clear, and the isolated eggs of *F. hepatica* were used in the EHT.

*F. hepatica* eggs for the FECRT were obtained using the same method as in the EHT, except that 10-g samples were collected and examined individually for each animal on all sampling days. After cleaning the suspension and removing the supernatant, the sediment was transferred to a petri dish, where the eggs were counted under a microscope at magnifications of 10–40×.

### 2.3. FECRT

The FECRT was used to determine the efficacy of ABZ in vivo. A faecal egg count (FEC) of *F. hepatica* eggs per 10-g sample was conducted, and the percentage reduction of eggs was determined using the formula [35]:

(1) $$\mathrm{Efficacy}=\left[\frac{\left(\mathrm{FEC}\;D0-\mathrm{FEC}\;D\;14\right)}{\left(\mathrm{FEC}\;D\;0\right)}\right]\times \;100$$

The formula FECRT (%) = 100 × (1 − [T ÷ C]), recommended by the World Association for the Advancement of Veterinary Parasitology (WAAVP) [36,37], was also used to evaluate efficacy, where T is the arithmetic mean EPG in the treated group 14 days after treatment and C is the arithmetic mean EPG in the control group at D14. A lack of drug efficacy was suspected if the percent reduction was <90% [11].

### 2.4. Egg Hatch Test (EHT)

Performing the EHT was based on a study conducted by Alvarez et al. [33], with some modifications. The most important modification was that *F. hepatica* eggs were isolated from faecal samples instead of gallbladders.

The EHT was performed in two versions. In the first version, fluke eggs (200/1 mL of water) were placed in the tubes and incubated at 25 °C in the dark for 12 h (version 12H) at final ABZ concentrations of 0.05, 0.5 and 5.0 μM. Ten microlitres of methanol were used for the negative control. The eggs were then washed three times with tap water to remove the anthelmintic. The content from the tubes was placed in 24-well plates with 1 mL of water and incubated again in the dark at 25 °C for 15 d. The plates were then exposed to light, and 10 μL of 10% buffered formalin was added to each well after 2 h.

Approximately 80–100 eggs were evaluated in each well under an optical microscope (40× magnification). The term “hatched eggs” included embryonated eggs containing developed miracidia. Ovicidal activity was determined for each concentration using the formula:

(2) $$\mathrm{Ovicidal}\;\mathrm{activity}=\frac{\;\%\;\mathrm{eggs}\;\mathrm{hatched}\;\mathrm{in}\;\mathrm{control}-\%\;\mathrm{eggs}\;\mathrm{hatched}\;\mathrm{after}\;\mathrm{drug}\;\mathrm{incubation}}{\%\;\mathrm{eggs}\;\mathrm{hatched}\;\mathrm{in}\;\mathrm{control}}\times 100$$

The second version of the test was prepared similarly, except that the anthelmintic was not removed after 12 h, and the eggs were exposed to the ABZ for 15 d (version 15D). Five replicates were used for each concentration in each version.

### 2.5. Statistical Analysis

An unpaired *t*-test was applied to evaluate the differences between FEC before and after treatment. Statistical significance was set at *p* < 0.05. Analyses were conducted using Graph Pad Prism 9.1.0 (GraphPad Software, San Diego, CA, USA).

## 3. Results

### 3.1. FECRT

The arithmetic mean FECs in the control and treated groups on D0 and D14 after therapy are presented in Table 1. All animals in the study were positive for *F. hepatica* on D0. The number of *F. hepatica* eggs in 10 g of faeces varied from 10 to 95, and mean FEC was comparable for both groups at the beginning of the experiment. The ratio of positive animals on D14 was 7/10 in the treated group, and all 10 animals remained positive in the control group. Diarrhoea was observed in several animals in both groups. ABZ efficacy ranged from 77 to 81.8% depending on the method of calculation, indicating a significant effect of the treatment (*p* < 0.05). Both methods are presented in Table 1.

### 3.2. EHT

Mean ovicidal activity at all concentrations for both versions of the test is summarised in Table 2. The mean (93.60%) was highest in version 15D at a concentration of 5.0 μM ABZ. The mean at the same concentration was slightly lower (84.11%) in version 12H. Similarly, mean ovicidal activity at the other two concentrations was higher in version 15D.

The mean percentage of eggs prevented from hatching was most comparable with the in vivo data at a concentration of 0.5 μM in both versions of the test. Hatching was higher at all concentrations in version 12H. Differences in mean percent hatching between the two versions of the test are presented in Table 3.

## 4. Discussion

The main goal of this study was to identify the efficacy of ABZ on a cattle farm with a long-term use of ABZ and regular presence of fasciolosis. Interpretating the results was quite complicated due to the absence of standardised protocols. Our results confirmed a reduced efficacy (77.0–81.8%) of ABZ against *F. hepatica* based on the WAAVP guidelines mainly for gastrointestinal parasites. An efficacy of 90% is commonly used for trematodes [11], even though the FECRT has not yet been standardised. Fairweather et al. [1] reported that efficacies of 71–90% may be considered sufficient for drugs such as ABZ or oxyclozanide. Even if the percent reduction does not exceed 90%, ABZ may still reduce fluke burden to a level that will be beneficial from the points of view of economics and animal health [38,39].

Many factors may affect the efficacy of an anthelmintic, such as incorrect dosing due to inaccurate weighing, improper application and the metabolic status of treated animals, where pathological changes to the liver can affect the bioavailability of anthelmintics [40]. These factors could subsequently affect the results of an FECRT. The FECRT was used as the only method in 41% of studies about the diagnosis of TCBZ resistance [1], despite the well-known limitations.

In the absence of recommended thresholds and standardised protocols, we also cannot immediately use the term “resistance” when drug efficacy is <90%. The recommendation of diagnostics using a minimal number of two methods should be part of new standardised protocols for determining AR and anthelmintic efficacy against trematodes.

The EHT was the second “confirmation” method in our study. Protocols for test preparation differed mainly in the source of eggs (faeces, gallbladder) and amount of time the eggs were exposed to the drug (12H, 15D). An interval of 12 h has been described as an approximate time for exposing eggs to an anthelmintic after in vivo treatment [33]. Ceballos et al. [15] confirmed the importance of incubation period for a highly resistant isolate of *F. hepatica*, where ovicidal activity increased from 1.7% (12H) to susceptible status (92.6%) (15D). Similarly, we recorded higher mean ovicidal activities, from 9 to 22%, at all concentrations in version 15D. This result may have been influenced by the different egg sources used between the two studies. Canevari et al. [32] suggested that an ABZ concentration of 0.5 μM could serve as a cut off value for evaluating in vitro resistance. These authors found that the tested drug was effective when ovicidal activity at this concentration was >70%, resistance was suspected between 40 and 70% and activities <40% indicated resistance.

Our results also highlight the relevance of incubation time, where ovicidal activity at an ABZ concentration of 0.5 μM differed between EHT versions 12H (65.40%) and 15D (79.18%). The 12H results indicated suspected resistance, and ovicidal activity increased to the susceptible level after 15 d. Mean hatching at a concentration of 0.5 μM in both versions, though, was consistent with the in vivo (FECRT) data. Mean hatching at a concentration of 0.5 μM was 26.20 and 14.16% for the 12H and 15D versions, respectively. The results from version 15D may be slightly overestimated, so we can assume that the efficacy of ABZ ranged approximately from 70 to 80%.

Comparing results from the EHT with a controlled efficacy test will be more suitable and accurate, as performed by Ceballos et al. [15]. This method is unfortunately difficult to perform on local farms, mainly due to economic reasons. Similarly, Arafa et al. [41] compared hatching at ABZ concentrations between 0.0002 and 2 μg/mL and the percentage reduction of faecal egg counts two weeks after treatment in naturally infected cattle. These authors observed differences between the percent reduction of eggs (73.7%) and mean hatching at the two highest concentrations in an EHT (7.4 and 5.6%). 

The discrepancies between the in vivo and in vitro results in our study may have been due to the limited efficacy of ABZ in immature flukes, where eggs identified after two weeks may have been from previously juvenile flukes. Monitoring hatching at individual concentrations was a suitable way to avoid misinterpreting the in vivo results. Hatching in sensitive isolates will decrease with increased concentrations but hatching in resistant isolates will be similar for all concentrations [42].

The results of an EHT can be influenced by several factors. Percentage hatching can be affected by the source of eggs used in the preparation of the test. Most studies that have focused on the standardisation of the EHT, however, used fluke eggs isolated from the gallbladder [15,32,33]. This source is more suitable for sample purity, but faecal samples will have to be primarily used in comprehensive surveys of a larger number of farms, because the slaughter of animals is not always possible. Each purification of a sample during sedimentation leads to the loss of eggs. Obtaining both sample purity and the required number of eggs (80–100) per well can therefore sometimes be difficult on farms with lower intensities of infection. Róbles-Peréz et al. [42] reported that impurities from faecal samples could affect hatching rates in an EHT. The authors could nevertheless distinguish between susceptible and resistant isolates in experimentally infected sheep. Novobilský et al. [18] successfully used the EHT with pooled faecal samples for detecting reduced ABZ efficacy on a sheep farm in Sweden.

Length of egg storage is another factor that can affect the results of the test. Ceballos et al. [15] recommended a maximum storage time of 2 months after the collection of eggs. These authors observed a significant reduction in ovicidal activity in eggs stored for a longer period (6 months).

We performed the EHT only using the field strain of *F. hepatica* isolated on the farm, which was a possible limitation of this study. The most appropriate approach would be to check the suitability of our methodology using known susceptible and resistant isolates of *F. hepatica* and comparing the results with field strains. This approach unfortunately could not be applied when we performed our study.

In conclusion, more studies of naturally infected animals are needed for developing standardised guidelines. The EHT in our study was prepared using pooled faecal samples, which was a useful method for the detection of efficacy and AR in *F. hepatica.* Comparison with the in vivo results confirmed that 0.5 μM ABZ could serve as a useful cut-off concentration for the determination of resistance, as reported by Canevari et al. [32] and Ceballos et al. [15]. The concentration of 0.5 μM ABZ could also serve as a reliable indicator of in vivo drug efficacy, but a survey of a larger number of farms is needed for confirmation. The evaluation of the two variants of the EHT indicated that the results from version 15D were slightly overestimated. The version where fluke eggs were exposed to the drug for 12 h was the most appropriate EHT variant for detecting AR in *F. hepatica.*

## Figures and Tables

**Table 1:** Mean FEC and percent reduction of *F. hepatica* eggs for the two methods of calculation.

ABZ ^1^ 7.5 mg/kg bw	P/A ^5^	Mean FEC ^2^	SD ^4^	P/A ^5^	Mean FEC	SD ^4^	FECRT (%) ^3^	
		D0			D14		Foreyt [35]	Coles et al. [36]
Treated group	10/10	33.52 *	25.81	7/10	7.40 *	7.04	77.00	81.80
Control group	10/10	45.31	23.14	10/10	40.50	21.64		

^1^ Albendazole; ^2^ Faecal egg count; ^3^ Faecal egg count reduction test; ^4^ Standard deviation (±); ^5^ Number of *F. hepatica* positive (P) animals/number of animals (A) in the group; * Indicates a significant difference (*p* < 0.05).

**Table 2:** Comparison of the mean ovicidal activity (%) of ABZ between the two versions of the EHT ^2^.

Ovicidal Activity (%)				
ABZ (μM) ^1^	12H		15D	
	Mean	SD ^3^	Mean	SD ^3^
0.05	37.34	22.38	59.99	17.34
0.5	65.40	16.36	79.18	6.45
5.0	84.11	11.94	93.60	4.72

^1^ Albendazole; ^2^ Egg hatch test; ^3^ Standard deviation (±).

**Table 3:** Comparison of mean percent hatching at different concentrations between the two versions of the EHT.

Mean Hatching (%)				
	12H		15D	
ABZ (μM) ^1^	Mean	SD ^2^	Mean	SD ^2^
control	78.80	8.79	81.00	9.14
0.05	47.60	14.02	31.80	12.56
0.5	26.20	11.28	14.16	8.53
5.0	12.10	8.83	5.40	4.17

^1^ Albendazole; ^2^ Standard deviation (±).

## Data Availability

Data are available on request.

## References

[1] Fairweather I., Brennan G.P., Hanna R.E.B., Robinson M.W., Skuce P.J. (2020). Drug resistance in liver flukes. Int. J. Parasitol. Drugs Drug Resist..

[2] Fairweather I. (2005). Triclabendazole: New skills to unravel an old(ish) enigma. J. Helminthol..

[3] Haseeb A.N., El-Shazly A.M., Arafa M.A., Morsy A.T. (2002). A Review on fascioliasis in Egypt. J. Egypt Soc. Parasitol..

[4] Mas Coma S., Bargues M.D., Valero M.A. (2018). Human fascioliasis infection sources, their diversity, incidence factors, analytical methods and prevention measures. Parasitology.

[5] Winkelhagen A.J., Mank T., de Vries P.J., Soetekouw R. (2012). Apparent triclabendazole-resistant human Fasciola hepatica infection, the Netherlands. Emerg. Infect. Dis..

[6] Boray J.C., Crowfoot P.D., Strong M.B., Allison J.R., Schellenbaum M., Von Orelli M., Sarasin G. (1983). Treatment of immature and mature *Fasciola hepatica* infections in sheep with triclabendazole. Vet. Rec..

[7] Overend D.J., Bowen F.L. (1995). Resistance of *Fasciola hepatica* to triclabendazole. Aust. Vet. J..

[8] Kelley J.M., Elliott T.P., Beddoe T., Anderson G., Skuce P., Spithill T.W. (2016). Current threat of triclabendazole resistance in *Fasciola hepatica*. Trends Parasitol..

[9] McMahon C., Edgar H.W.J., Hanna R.E.B., Ellison S.E., Flanagan A.M., McCoy M., Kajugu P.-E., Gordon A.W., Irwin D., Barley J.E. (2016). Liver fluke control on sheep farms in Northern Ireland: A survey of changing management practices in relation to disease prevalence and perceived triclabendazole resistance. Vet. Parasitol..

[10] McKellar Q., Scott E. (1990). The benzimidazole anthelmintic agents—A review. J. Vet. Pharmacol. Ther..

[11] Fairweather I., Boray J.C. (1999). Fasciolicides: Efficacy, actions, resistance and its management. Vet. J..

[12] Coles G.C., Stafford K.A. (2001). Activity of oxyclozanide, nitroxynil, clorsulon and albendazole against adult triclabendazole-resistant Fasciola hepatica. Vet. Rec..

[13] Mamani W., Condori R. (2009). Anthelmintic resistance (*Fasciola hepatica*) in sheep against albendazole and triclabendazole, La Paz-Bolivia. Rev. Investig. Vet. Perú (RIVEP).

[14] Sanabria R., Ceballos L., Moreno L., Romero J., Lanusse C., Alvarez L. (2013). Identification of a field isolate of *Fasciola hepatica* resistant to albendazole and susceptible to triclabendazole. Vet. Parasitol..

[15] Ceballos L., Canton C., Pruzzo C., Sanabria R., Moreno L., Sanchis J., Suarez G., Ortiz P., Fairweather I., Lanusse C. (2019). The egg hatch test: A useful tool for albendazole resistance diagnosis in *Fasciola hepatica*. Vet. Parasitol..

[16] Alvarez-Sanchez M.A., Mainar-Jaime R.C., Perez-Garcia J., Rojo-Vazquez F.A. (2006). Resistance of *Fasciola hepatica* to triclabendazole and albendazole in sheep in Spain. Vet. Rec..

[17] Martinez-Valladares M., Cordero-Perez C., Rojo-Vazquez F.A. (2014). Efficacy of an anthelmintic combination in sheep infected with *Fasciola hepatica* resistant to albendazole and clorsulon. Exp. Parasitol..

[18] Novobilsky A., Solis N.A., Skarin M., Hoglund J. (2016). Assessment of flukicide efficacy against *Fasciola hepatica* in sheep in Sweden in the absence of a standardised test. Int. J. Parasitol. Drugs Drug Resist..

[19] Elitok B., Elitok O.M., Kabu M. (2006). Field trial on comparative efficacy of four fasciolicides against natural liver fluke infection in cattle. Vet. Parasitol..

[20] Chavez A., Sanchez L., Arana C., Suarez F. (2012). Resistance to anthelmintics and prevalence of bovine fasciolosis in dairy farms in Juaja, Peru. Rev. Investig. Vet. Perú (RIVEP).

[21] Shokier K.M., Aboelhadid S.M., Waleed M.A. (2013). Efficacy of five anthelmintics against a natural *Fasciola* species infection in cattle. Beni-Suef Univ. J. Basic Appl. Sci..

[22] Nzalawahe J., Hannah R., Kassuku A.A., Stothard J.R., Coles G., Eisler M.C. (2018). Evaluating the effectiveness of trematocides against *Fasciola gigantica* and amphistomes infections in cattle, using faecal egg count reduction tests in Iringa Rural and Arumeru Districts, Tanzania. Parasites Vectors.

[23] Wood I.B., Amaral N.K., Bairden K., Duncan J.L., Kassai T., Malone J.B., Pankavich J.A., Reinecke R.K., Slocombe O., Taylor S.M. (1995). World Association for the Advancement of Veterinary Parasitology (W.A.A.V.P.) second edition of guidelines for evaluating the efficacy of anthelmintics in ruminants (bovine, ovine, caprine). Vet. Parasitol..

[24] Mitchell G.B.B., Maris L., Bonniwell M.A. (1998). Triclabendazole-resistant liver fluke in Scottish sheep. Vet. Rec..

[25] Flanagan A., Edgar H.W.J., Gordon A.W., Hanna R.E.B., Brennan G.P., Fairweather I. (2011). Comparison of two assays, a faecal egg count reduction test (FECRT) and a coproantigen reduction test (CRT), for the diagnosis of resistance to triclabendazole in *Fasciola hepatica* in sheep. Vet. Parasitol..

[26] Mezo M., González-Warleta M., Carro C., Ubeira F.M. (2004). An ultrasensitive capture ELISA for detection of Fasciola hepatica coproantigens in sheep and cattle using a new monoclonal antibody (M3). J. Parasitol..

[27] Gordon D.K., Zadoks R.N., Stevenson H., Sargison N.D., Skuce P.J. (2012). On farm evaluation of the coproantigen ELISA and coproantigen reduction test in Scottish sheep naturally infected with Fasciola hepatica. Vet. Parasitol..

[28] Brockwell Y.M., Elliott T.P., Anderson G.R., Stanton R., Spithill T.W., Sangster N.C. (2014). Confirmation of *Fasciola hepatica* resistant to triclabendazole in naturally infected Australian beef and dairy cattle. Int. J. Parasitol. Drugs Drug Resist..

[29] Elliott T.P., Kelley J.M., Rawlin G., Spithill T.W. (2015). High prevalence of fasciolosis and evaluation of drug efficacy against *Fasciola hepatica* in dairy cattle in the Maffra and Bairnsdale districts of Gippland, Victoria, Australia. Vet. Parasitol..

[30] Hanna R.E.B., McMahon C., Ellison S., Edgar H.W., Kajugu P.E., Gordon A., Irwin D., Barley J.P., Malone F.E., Brennan G.P. (2015). *Fasciola hepatica*: A comparative survey of adult fluke resistance to triclabendazole, nitroxynil and closantel on selected upland and lowland sheep farms in Northern Ireland using faecal egg counting, coproantigen ELISA testing and fluke histology. Vet. Parasitol..

[31] Coles G.C., Briscoe M.G. (1978). Benzimidazoles and fluke eggs. Vet. Rec..

[32] Canevari J., Ceballos L., Sanabria R., Romero J., Olaechea F., Ortiz P., Cabrera M., Gayo V., Fairweather I., Lanusse C. (2014). Testing albendazole resistance in *Fasciola hepatica*: Validation of an egg hatch test with isolates from South America and the United Kingdom. J. Helminthol..

[33] Alvarez L., Moreno G., Moreno L., Ceballos L., Shaw L., Fairweather I., Lanusse C. (2009). Comparative assessment of albendazole and triclabendazole ovicidal activity on *Fasciola hepatica* eggs. Vet. Parasitol..

[34] Graham-Brown J., Williams D.J.L., Skuce P., Zadoks R.N., Dawes S., Swales H., Van Dijk J. (2019). Composite *Fasciola hepatica* faecal egg sedimentation test for cattle. Vet. Rec..

[35] Foreyt W.J. (1988). Efficacy and safety of albendazole against experimentally induced Fasciola hepatica infections in goats. Vet. Parasitol..

[36] Coles G.C., Bauer C., Borgsteede F.H.M., Geerts S., Klei T.R., Taylor M.A., Waller P.J. (1992). World Association for the Advancement of Veterinary Parasitology (W.A.A.V.P.) methods for the detection of anthelmintic resistance in nematodes of veterinary importance. Vet. Parasitol..

[37] Coles G.C., Jackson F., Pomroy W.E., Prichard R.K., von Samson-Himmelstjerna G., Silvestre A., Taylor M.A., Vercruysse J. (2006). The detection of anthelmintic resistance in nematodes of veterinary importance. Vet. Parasitol..

[38] Fairweather I. (2011). Reducing the future threat from (liver) fluke: Realistic prospect or quixotic fantasy?. Vet. Parasitol..

[39] Forbes A.B. (2013). Liver fluke control in cattle: Why, when and how?. Cattle Pract..

[40] Fairweather I. (2011). Liver fluke isolates: A question of provenance. Vet. Parasitol..

[41] Arafa W.M., Shokeir K.M., Khateib A.M. (2015). Comparing an in vivo egg reduction test and in vitro egg hatching assay for different anthelmintics against *Fasciola* species, in cattle. Vet. Parasitol..

[42] Robles-Perez D., Martinez-Perez J.M., Rojo-Vazquez F.A., Martinez-Valladares M. (2014). Development of an egg hatch assay for the detection of anthelmintic resistance to albendazole in *Fasciola hepatica* isolated from sheep. Vet. Parasitol..

